# Supplementary material for: The Combination of Upconversion Nanoparticles and Perovskite Quantum Dots with Temperature-Dependent Emission Colors for Dual-Mode Anti-Counterfeiting Applications
Source: Nanomaterials (Basel). 2023 Dec 8;13(24):3102. doi: 10.3390/nano13243102 (PMC10745397; doi:10.3390/nano13243102)
Supplement: Supplementary file 1 [file nanomaterials-13-03102-s001.zip › nanomaterials-2730776-supplementary.pdf]

Supporting Information

The Combination of Upconversion Nanoparticles and Perovskite Quantum Dots with Temperature-Dependent Emission Colors for Dual-Mode Anti-Counterfeiting Applications

Qun Zhang [1], Yuefeng Gao [2,*], Lihong Cheng [1,*], You Li [1], Sai Xu [1,*] and Baojiu Chen [1]

[1] School of Science, Dalian Maritime University, Dalian 116026, China; qunmay529@sina.com (Q.Z.); lyzhx0519@163.com (Y.L.); bjchen@dlmu.edu.cn (B.C.)

[2] Marine Engineering College, Dalian Maritime University, Dalian 116026, China

* Correspondence: gaoyuefeng@dlmu.edu.cn (Y.G.); clh6833@dlmu.edu.cn (L.C.); xsjlu@126.com (S.X.)

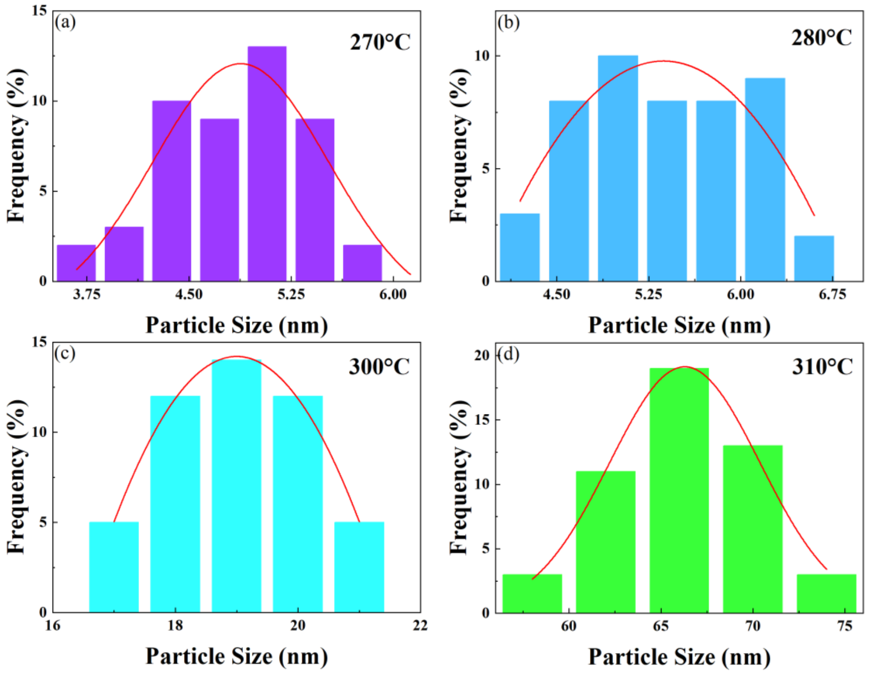

Figure S1. Corresponding particle size distributions of $NaGdF_4$: Yb/Tm synthesized at (a) 270 °C, (b) 280 °C, (c) 300 °C, (d) 310 °C.

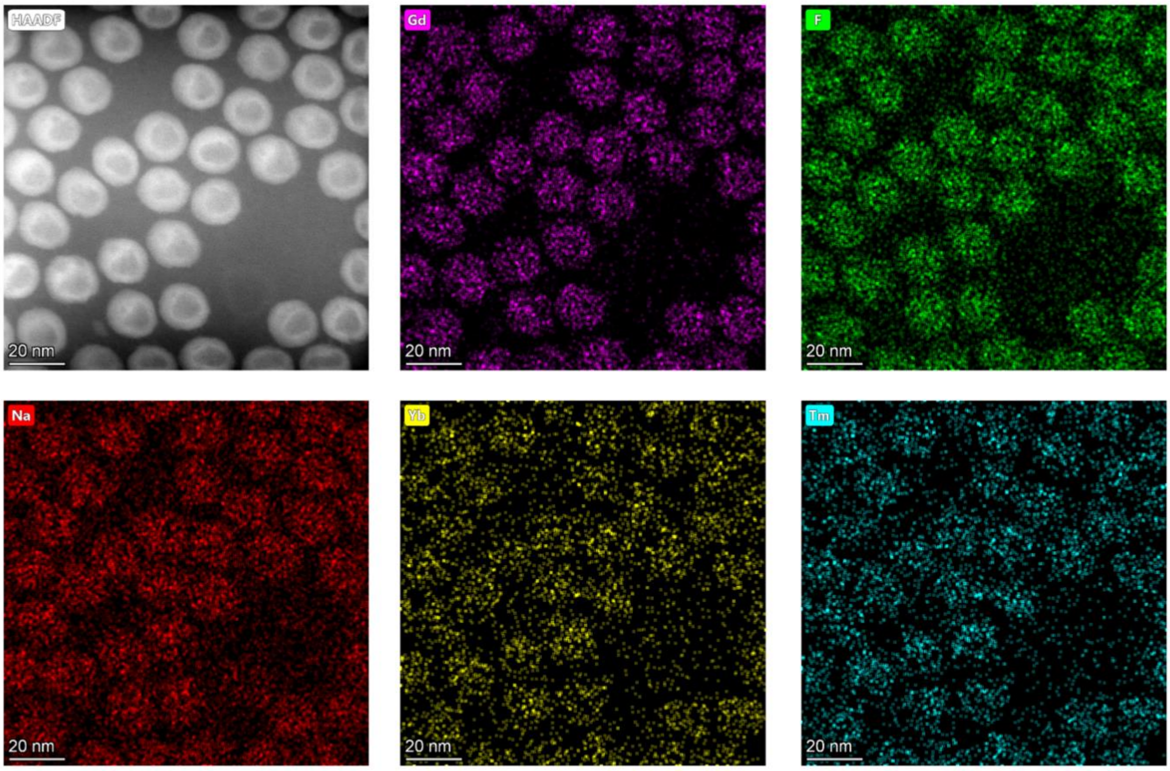

Figure S2. EDS mapping images of UCNPs synthesized at 300 ℃

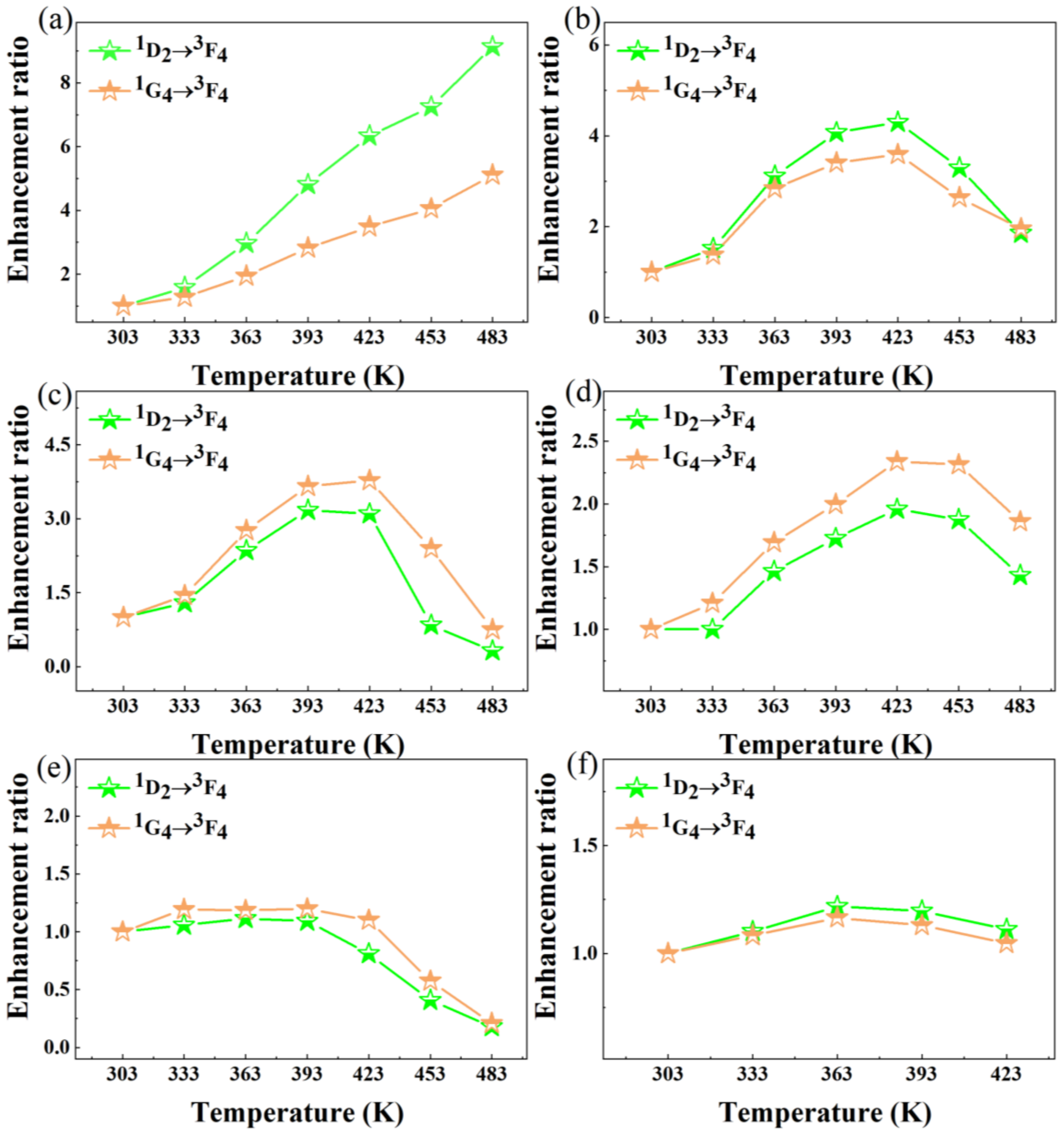

Figure S3. Integral luminescence intensities of $^{1}D_{2}$-$^{3}F_{4}$ and $^{1}G_{4}$-$^{3}F_{4}$ of $NaGdF_4$: Yb/Tm UCNPs with size of (a)4.94 nm, (b)5.43 nm, (c)18.98 nm, (d)66.09 nm. Integral luminescence intensities of $^{1}D_{2}$-$^{3}F_{4}$ and $^{1}G_{4}$-$^{3}F_{4}$ of (e) core @ active shell UCNPs; (f) core @ inert shell UCNPs.

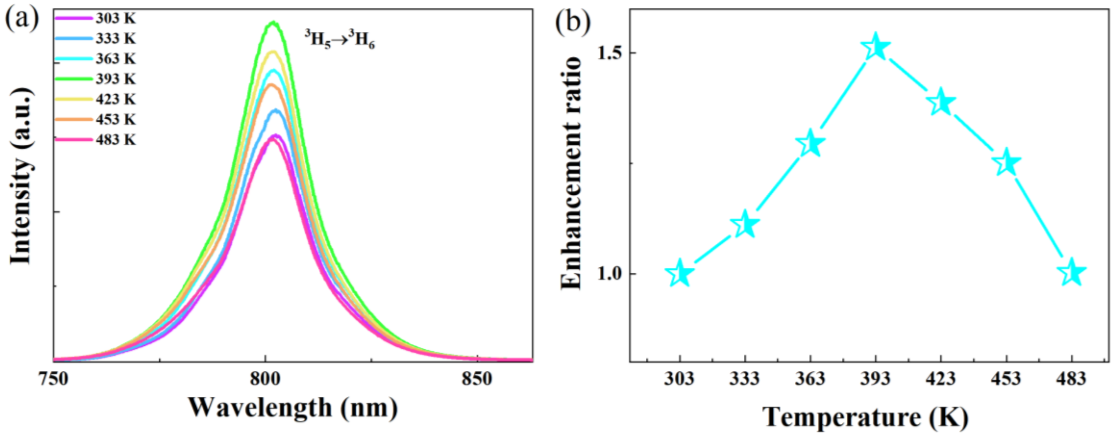

Figure S4. (a) Temperature-dependent infrared emission spectra of $NaGdF_4$: Yb/Tm UCNPs synthesized at 300°C; (b) Integral luminescence intensities of $^3H_5 \to ^3H_6$ of $NaGdF_4$: Yb/Tm UCNPs synthesized at 300°C
